# Prevalence and risk characteristics of COVID-19 in outpatients: A cross-sectional study of New York-area clinics

**DOI:** 10.25122/jml-2021-0087

**Published:** 2021

**Authors:** Chanapong Rojanaworarit, Douglas Charles Lambert, Joseph Conigliaro, Eun Ji Kim

**Affiliations:** 1. Department of Health Professions, School of Health Professions and Human Services, Hofstra University, Hempstead, NY, USA; 2. Department of General Internal Medicine, Northwell Health, Great Neck, NY, USA; 3. Section of Obesity Medicine, Northwell Health, Great Neck, NY, USA; 4. Zucker School of Medicine at Hofstra/Northwell, Hempstead, NY, USA; 5. Feinstein Institutes for Medical Research, Great Neck, NY, USA

**Keywords:** COVID-19, outpatient, race, ethnicity, smoking, non-communicable disease

## Abstract

Outpatients can be at heightened risk of COVID-19 due to interaction between existing non-communicable diseases in outpatients and infection with severe acute respiratory syndrome coronavirus 2 (SARS-CoV-2). This study measured the magnitude of COVID-19 prevalence and explored related risk characteristics among adult outpatients visiting medicine clinics within a New York state-based tertiary hospital system. Data were compiled from 63,476 adult patients visiting outpatient medicine clinics within a New York-area hospital system between March 1, 2020, and August 28, 2020. The outcome was a clinical diagnosis of COVID-19. Crude and adjusted prevalence ratios (PR) of a COVID-19 were analyzed using univariable and multivariable Poisson regression with robust standard errors. The prevalence of COVID-19 was higher among these outpatients (3.0%) than in the total population in New York State (2.2%) as of August 28, 2020. Multivariable analysis revealed adjusted prevalence ratios significantly greater than one for male sex (PR=1.10), age 40 to 64 compared to <40 (PR=1.19), and racial/ethnic minorities in comparison to White patients (Hispanic: PR=2.76; Black: PR=1.89; and Asian/others: PR=1.56). Nonetheless, factors including the advanced age of ≥65 compared to <40 (PR=0.69) and current smoking compared to non-smoking (PR=0.60) were related to significantly lower prevalence. Therefore, the prevalence of COVID-19 in outpatients was higher than that of the general population. The findings also enabled hypothesis generation that routine clinical measures comprising sex, age, race/ethnicity, and smoking were candidate risk characteristics of COVID-19 in outpatients to be further verified by designs capable of assessing temporal association.

## Introduction

The profound effect of severe acute respiratory syndrome coronavirus 2 (SARS-CoV-2) infection has resulted from the intersection of a highly infectious virus with a pre-existing pandemic of non-communicable diseases (NCDs) in adults and the elderly and from potentially greater risk of viral exposure among vulnerable demographic groups. Individuals with NCDs, such as hypertension, diabetes mellitus, and chronic obstructive pulmonary disease (COPD), have been indicated to be at heightened risk of SARS-CoV-2 infection and severe coronavirus disease 2019 (COVID-19) [1–3]. Analyses in the US general population and international patients with COVID-19 unveiled greater infection rates and mortality due to COVID-19 among racial/ethnic minorities [4, 5]. The disproportionate COVID-19 occurrence and outcome across different races and ethnicities may be attributed to greater comorbidity rates in certain racial and ethnic groups [6]. Therefore, comorbidity and differences in social vulnerabilities across demographic groups appear to play important roles in the etiology of COVID-19 and its adverse clinical consequences. Nonetheless, studies devoted to understanding these potential risk characteristics of COVID-19 in the domain of outpatients who have an array of existing non-communicable diseases and are plausibly at increased risk of the infection and adverse outcomes are still scarce. Hospital-based studies previously examining these risk characteristics have been mainly conducted in inpatient populations, precluding the generalizability of findings to the outpatients who should be the targeted group for early prevention before infection and severe consequences of COVID-19. Apart from comorbidity, the role of smoking as a risk characteristic of COVID-19 has been investigated among inpatients and revealed inconsistent results [7–8]. While smokers hospitalized with COVID-19 have greater odds of disease progression [7], consistently low prevalences of smoking have been observed among patients hospitalized with COVID-19 in several studies [8]. In an outpatient setting, smokers have been shown to have lower odds of SARS-CoV-2 test positive results [9]. Investigation in outpatient settings across different countries would provide additional evidence for evaluating the suggested relationship.

Since comorbidity among outpatients may impose a high risk of SARS-CoV-2 infection and severe COVID-19 if hospitalized [1–3]. Determining clinical risk characteristics of COVID-19 from routinely available data of these patients would provide preliminary information regarding risk profiles that clinicians can practically consider for early prevention and counseling of these vulnerable patients from the infection and adverse consequences during this unprecedented situation. Furthermore, anticipated findings of the relationship between clinical characteristics and COVID-19 would also enable hypothesis generation regarding candidate risk characteristics of COVID-19 in outpatients. The hypothesis could be further verified by study designs capable of assessing temporal association that can be useful for the development of a future clinical guideline for COVID-19 prevention in outpatients during and beyond this pandemic. Therefore, this study measured the magnitude of COVID-19 prevalence and explored related clinical risk characteristics among adult outpatients visiting medicine clinics within a New York state-based tertiary hospital system.

## Material and Methods

### Patient domain

Study patients included adult outpatients aged 18 years old and above, visiting Northwell Health ambulatory family medicine and internal medicine clinics from March 1, 2020, to August 28, 2020. Northwell Health is a large, US-based tertiary hospital system with 23 hospitals and approximately 800 clinics in New York City and surrounding suburban areas. Patients’ clinical data attending 28 medicine clinics were collected for this study. Data from each patient’s most recent outpatient visit were extracted to provide the most updated information regarding clinical characteristics and COVID-19 diagnosis.

### Measures

Patient demographics, comorbidities, risk behavior, and COVID-19 diagnosis were obtained from the outpatient electronic health record (EHR; Allscripts, Chicago, IL) of Northwell Health. The outcome was a clinical diagnosis of COVID-19, as indicated by an ICD-10 diagnosis code (U07.1). Demographic characteristics included sex, age, and race/ethnicity. Comorbidities included hypertension, diabetes, coronary artery disease, congestive heart failure, and COPD. Smoking status was classified into three categories: non-, former, and current smokers. Clinic locations were also collected for covariate adjustment during regression analysis.

### Statistical analysis

Descriptive statistics were used to summarize the characteristics of the study patients. The two-sample t-test was employed to compare the mean ages of outpatients by COVID-19 status. Chi-squared tests were used to compare the proportions of the outcome among categories of each categorical explanatory variable. Univariable and multivariable Poisson regression with robust standard errors were applied to directly estimate crude and adjusted COVID-19 prevalence ratios [10, 11].

## Results

The majority of the 63,476 outpatients in this study were female (60.5%) and had an average age of 58 years. Merely 11.7 percent of patients were black, while most of the patients (67.3%) were white. About 9% of the patients were active smokers. Hypertension was present in about half of the outpatients (47.2%). Diabetes mellitus was diagnosed in about 18% of the total patients. Coronary artery disease and congestive heart failure were found in 19.5% and 2.7% of the total patients. Outpatients with COPD accounted for 6.3 percent of the total. There were 28,902 patients (45.5%) without the aforementioned five non-communicable diseases (Table 1).

**Table 1.** Characteristics of outpatients by COVID-19 status.

**Chracteristics**	**Total** **n (%) †**	**COVID-19**		**P-value**
		**No** **n (%) ‡**	**Yes** **n (%) ‡**	
**All outpatients**	63,476	61,589 (97.0)	1,887 (3.0)	
**Sex**				
Female	38,382 (60.5)	37,243 (97.0)	1,139 (3.0)	0.923 *
Male	25,094 (39.5)	24,346 (97.0)	748 (3.0)	
**Age (year)**				
Mean±SD	58.0±17.2	58.1±17.3	54.9±15.1	<0.001 **
Min. – Max.	18–103	18–103	19–100	
<40	10,762 (17.0)	10,432 (96.9)	330 (3.1)	
40–64	28,270 (44.5)	27,216 (96.3)	1,054 (3.7)	
≥65	24,444 (38.5)	23,941 (97.9)	503 (2.1)	
**Race/ethnicity**				
White	42,728 (67.3)	41,864 (98.0)	864 (2.0)	<0.001 *
Black	7,447 (11.7)	7,070 (94.9)	377 (5.1)	
Asian / others	8,219 (13.0)	7,907 (96.2)	312 (3.8)	
Hispanic	5,082 (8.0)	4,748 (93.4)	334 (6.6)	
**Smoking status**				
Non-smoker	39,402 (62.1)	38,110 (96.7)	1,292 (3.3)	<0.001 *
Former smoker	18,560 (29.2)	18,066 (97.3)	494 (2.7)	
Current smoker	5,514 (8.7)	5,413 (98.2)	101 (1.8)	
**Hypertension**				
No	33,545 (52.8)	32,520 (96.9)	1,025 (3.1)	0.193 *
Yes	29,931 (47.2)	29,069 (97.1)	862 (2.9)	
**Diabetes mellitus**				
No	51,992 (81.9)	50,485 (97.1)	1,507 (2.9)	0.019 *
Yes	11,484 (18.1)	11,104 (96.7)	380 (3.3)	
**Coronary artery disease**				
No	57,437 (90.5)	55,704 (97.0)	1,733 (3.0)	0.042 *
Yes	6,039 (9.5)	5,885 (97.4)	154 (2.6)	
**Congestive heart failure**				
No	61,764 (97.3)	59,932 (97.0)	1,832 (3.0)	0.554 *
Yes	1,712 (2.7)	1,657 (96.8)	55 (3.2)	
**COPD**				
No	59,450 (93.7)	57,658 (97.0)	1,792 (3.0)	0.018 *
Yes	4,026 (6.3)	3,931 (97.6)	95 (2.4)	

SD – Standard deviation; Min. – Minimum; Max. – Maximum; COPD – Chronic obstructive pulmonary disease; † – Column percentage; ‡ – Row percentage; * – Chi-squared test; ** – Two-sample t-test with unequal variances.

COVID-19 was diagnosed in 1,887 patients (3.0%) of the total study participants. Patients with COVID-19 had a significantly lower average age than their counterparts. Although COVID-19 was found in only 2% of White patients, it was found in significantly higher proportions in Black (5.1%) and Hispanic (6.6%) patients. When compared to non-smokers, former and current smokers had significantly lower prevalences of COVID-19. COVID-19 was found to be significantly more prevalent in diabetic patients than in their non-diabetic counterparts. In contrast, COVID-19 prevalences were significantly lower in patients with coronary artery disease and COPD relative to outpatients who do not have these diseases (Table 1).

Multivariable analysis revealed a slightly greater COVID-19 prevalence in male outpatients than their female counterparts (adjusted PR=1.10). COVID-19 prevalence was also found to be slightly higher in patients aged 40 to 64 relative to those under 40 (adjusted PR=1.19). Patients over the age of 65, in contrast, had a significantly lower prevalence than those under the age of 40 (adjusted PR=0.69). Patients of Hispanic ethnicity, Black, Asian, and other races showed significantly higher prevalence estimates of COVID-19 than White patients; adjusted PRs: 2.76, 1.89, and 1.56, respectively. None of the non-communicable diseases presented in the outpatients was found to be significantly related to increased COVID-19 prevalence. Nonetheless, COVID-19 was less common in current smokers than in non-smokers (adjusted PR=0.6) (Table 2).

**Table 2.** Univariable and multivariable analyses of COVID-19 with the explanatory variables.

**Characteristics**	**COVID-19 cases n (%)**	**Univariable analysis**			**Multivariable analysis**		
		**PR †**	**95% CI**	**P-value**	**PR ‡**	**95% CI**	**P-value**
**Sex**							
Female	1,139 (3.0)	1.00	Reference	-	1.00	Reference	-
Male	748 (3.0)	1.00	0.92–1.10	0.923	1.10	1.01–1.21	0.038
**Age (year)**							
<40	330 (3.1)	1.00	Reference	-	1.00	Reference	-
40–64	1,054 (3.7)	1.21	1.08–1.37	0.002	1.19	1.05–1.35	0.008
≥65	503 (2.1)	0.67	0.59–0.77	<0.001	0.69	0.59–0.81	<0.001
**Race/ethnicity**							
White	864 (2.0)	1.00	Reference	-	1.00	Reference	-
Black or African American	377 (5.1)	2.50	2.22–2.82	<0.001	1.89	1.66–2.16	<0.001
Asian/other	312 (3.8)	1.88	1.65–2.13	<0.001	1.56	1.37–1.79	<0.001
Hispanic	334 (6.6)	3.25	2.87–3.68	<0.001	2.76	2.41–3.16	<0.001
**Smoking status**							
Non-smoker	1,292 (3.3)	1.00	Reference	-	1.00	Reference	-
Former smoker	494 (2.7)	0.81	0.73–0.90	<0.001	0.97	0.87–1.08	0.537
Current smoker	101 (1.8)	0.56	0.46–0.68	<0.001	0.60	0.49–0.74	< 0.001
**Hypertension**							
No	1,025 (3.1)	1.00	Reference	-	1.00	Reference	-
Yes	862 (2.9)	0.94	0.86–1.03	0.193	1.00	0.90–1.11	0.972
**Diabetes mellitus**							
No	1,507 (2.9)	1.00	Reference	-	1.00	Reference	-
Yes	380 (3.3)	1.14	1.02–1.28	0.019	1.02	0.91–1.15	0.685
**Coronary artery disease**							
No	1,733 (3.0)	1.00	Reference	-	1.00	Reference	-
Yes	154 (2.6)	0.85	0.72–0.99	0.043	1.04	0.88–1.24	0.641
**Congestive heart failure**							
No	1,832 (3.0)	1.00	Reference	-	1.00	Reference	-
Yes	55 (3.2)	1.08	0.83–1.41	0.553	1.15	0.88–1.51	0.305
**COPD**							
No	1,792 (3.0)	1.00	Reference	-	1.00	Reference	-
Yes	95 (2.4)	0.78	0.64–0.96	0.019	0.98	0.79–1.21	0.828

CI – confidence interval; COPD – chronic obstructive pulmonary disease; PR – prevalence ratio; † – Crude prevalence ratio estimated by Poisson regression with robust standard errors; ‡ – Adjusted prevalence ratio estimated by Poisson regression with robust standard errors adjusting for age, sex, race/ethnicity, smoking, hypertension, diabetes, coronary artery disease, congestive heart failure, chronic obstructive pulmonary disease, and clinic location.

## Discussion

As of August 28, 2020–the final date of data collection, the prevalence of COVID-19 among these outpatients was 3.0% greater than the overall population of New York State (2.2%) [12]. This evidence indicated the greater magnitude of COVID-19 in outpatients, with 54.5 percent having one or more of the underlying five non-communicable diseases. Regardless of statistical significance, PR interval estimates of 1.01 to 1.21 when comparing male to female outpatients revealed only a weak relationship between sex and COVID-19 infection (Table 2). Another US-based cross-sectional study of outpatient and inpatient combined data suggested a more pronounced relationship between male sex and COVID-19 positive diagnosis [13]. Research investigating the difference in mechanisms of SARS-CoV-2 infectivity in men and women is still lacking. However, sex differences in immune responses to viral antigens, in general, have been previously described [14]; which suggested the possibility of variation in SARS-CoV-2 infectivity by sex and might explain the difference in COVID-19 prevalence between male and female outpatients in this study. The finding of higher COVID-19 prevalence in younger outpatients (age <40) compared to the elderly ones (age ≥65) was consistent with another US-based cross-sectional study in the general population [15]. Higher COVID-19 transmission among younger individuals may be explained by the increased risk of contact with infected individuals due to activities like working and commuting. However, COVID-19 transmission among elderly outpatients should still be of considerable concern. Previous research has shown that most young individuals who became infected had a self-limiting infection and recovered well. At the same time, most of those who developed the more severe disease, needed admission to an intensive care unit, and eventually died were older [16]. The prevalence ratios greater than one observed among Hispanic, Black, and Asian/other race outpatients compared to White were consistent with a previously shown association of race/ethnicity minority status and COVID-19 infection risk [4]. Higher indices of social deprivation [17] and social vulnerabilities [18] in racial and ethnic minorities have been associated with a greater occurrence of COVID-19. In addition, outbreaks of COVID-19 have been reported extensively in job facilities with high proportions of workers of Hispanic ethnicity [19]. These findings suggested the role of disadvantaged socioeconomic background in racial and ethnic minorities as a risk indicator of COVID-19.

The lower COVID-19 prevalence among smokers aligned with previous findings of lower odds of SARS-CoV-2 test positivity among smokers compared to non-smokers [9]. A possible explanation was that outpatients who were active smokers, aware of the health risks posed by smoking and cautious about infection risk, may be more cautious of SARS-CoV-2 exposures. Alternatively, the previous finding showed that nicotine, a component of tobacco smoke, modulated multiple immune system components and affected angiotensin-converting enzyme 2 (ACE2) expression [20]. Since SARS-CoV-2 uses ACE2 as a receptor for cell entry [21], downregulation of ACE2 by smoking and nicotine [22] may mitigate the infectivity of SARS-CoV-2. Nonetheless, recent investigation contradicts the notion that smoking and nicotine decrease ACE2 [23]. Thus, more research is required to elucidate the role of smoking and the risk of SARS-CoV-2 transmission.

The strengths of our study include the large study size and the use of a clinical diagnosis, rather than test positivity alone, as our outcome. Although the cross-sectional study design does not establish a temporal relationship between patient clinical characteristics and the outcome, the findings are useful to enable hypothesis generation. Routine clinical measures comprising sex, age, race/ethnicity, and smoking were candidate risk characteristics of COVID-19 in outpatients and could be further verified by designs capable of assessing temporal association.

## Conclusions

The prevalence of COVID-19 in the outpatients was higher than that of the general population in New York State. Among outpatients visiting New York-area clinics, greater prevalences of COVID-19 were observed among male sex, age of 40 to 64 (compared to <40), and racial/ethnic minorities including Hispanic, Black, and Asian/other races (compared to White). Nonetheless, factors including the advanced age of ≥65 and current smoking were significantly related to lower prevalence.

## Acknowledgments

### Conflict of interest

The authors declare that there is no conflict of interest.

### Ethical approval

The Northwell Health Institutional Review Board determined the study to be exempt from review due to the use of pseudonymized data.

### Personal thanks

The authors would like to acknowledge Northwell Health for providing permission to obtain data from the outpatient electronic health record (EHR; Allscripts, Chicago, IL).

### Authorship

CR, DCL, JC and EJK conceived the idea for this paper. DCL carried out data extraction. CR and DCL conducted data cleaning, analysis and interpretation of results. All authors provided overview of analysis, supported manuscript writing, read and approved the final draft of the manuscript.
